# Physiologically Interpretable Photoplethysmography Features Associated With Peripheral Artery Disease

**DOI:** 10.1109/OJEMB.2026.3732080

**Published:** 2026-09-08

**Authors:** Ava J. Fascetti, Mustafa H. Naguib, Elsie G. Ross, Chris Longhurst, Pam R. Taub, Shamim Nemati, Edward J. Wang, Mattheus Ramsis

**Affiliations:** University of California San Diego La Jolla CA 92093 USA

**Keywords:** Ankle-brachial index, peripheral artery disease, photoplethysmography, pulse morphology, vascular physiology

## Abstract

*Objective:* Peripheral artery disease (PAD) is a common and frequently underdiagnosed cardiovascular condition. Photoplethysmography (PPG) is a widely available optical technique with strong potential to support peripheral vascular assessment, yet its feature-level relationship to PAD severity has not been systematically characterized at scale. This study aims to characterize physiologically informed, feature-level relationships between PPG pulse morphology and PAD severity using a large clinical dataset benchmarked against ankle-brachial index (ABI). *Results:* Using a large, multi-site clinical dataset consisting of 5,282 toe PPG waveforms from 2,599 unique patients, we systematically evaluated a broad set of PPG features. Features capturing waveform timing and shape exhibited consistent associations with disease severity, including pulse broadening and delayed waveform progression. In contrast, raw signal amplitude did not reliably associate with ABI. Feature associations were consistent across complementary feature classes, and exclusions did not introduce demographic bias. *Conclusions:* These findings show that PAD severity is reflected in coordinated changes in PPG pulse timing and shape, providing physiological context for waveform features. This work provides a feature-level reference framework for physiologically interpretable use of PPG in peripheral vascular assessment.

## I. Introduction

Peripheral artery disease (PAD), a circulatory condition in which atherosclerotic plaque limits blood flow to the extremities, affects nearly 12 million Americans and 200 million adults worldwide and is associated with increased risk of limb loss and major adverse cardiovascular events [1], [2], [3], [4]. Despite its prevalence and clinical consequences, PAD remains underdiagnosed, with many patients identified only after disease progression. The gold standard diagnosis of PAD is based on the ankle-brachial index (ABI), a noninvasive assessment that requires specialized equipment, trained personnel, and clinical time. The ABI is calculated for each leg as the ratio of systolic blood pressure measured at the ankle to that measured at the brachial artery. Although practical in theory, in reality, ABI screening is rarely used in routine care, with nearly 70% of primary care providers in the United States reporting never using ABI [5]. Limited screening contributes to delayed PAD diagnosis, particularly in underserved populations, where disparities in outcomes remain pronounced, causing substantial medical and financial burdens for patients and the US healthcare system [3], [6], [7], [8]. There exists an unmet clinical need for accessible PAD screening alternatives.

Photoplethysmography (PPG) is a noninvasive optical technique that measures changes in blood volume within a vascular bed and is widely available through wearable devices and smartphones [9]. Because PPG signals can capture the multifactorial endovascular sequelae of cardiovascular disease, including metabolic changes, endothelial dysfunction, and vascular tone [10], they have been used to detect several chronic cardiovascular conditions in prior work, including hypertension and atrial fibrillation [11], [12], [13]. The ubiquity and low cost of PPG make it an appealing modality for improving access to vascular health assessment. However, while PPG based approaches have shown promise, the relationship between specific waveform characteristics and PAD remains insufficiently understood.

Prior work has explored machine learning to detect PAD from PPG, but these approaches have relied on small cohorts, long signal durations, or models with limited interpretability and physiological insight [14], [15]. Other studies characterizing PPG morphology in vascular disease have depended on multi-site pulse comparisons rather than a single acquisition site suited to accessible screening [16], [17], [18]. As a result, it remains unclear which specific aspects of the single-site PPG pulse are altered by PAD and why. An improved physiological understanding of which PPG features carry PAD-related information is a prerequisite to building interpretable, generalizable assessment tools and understanding what any predictive model relies upon.

We previously reported initial associations between PPG features and ABI in this large clinical cohort [19], and have separately developed a predictive model that combines waveform and clinical features to detect ABI-defined PAD [20]. The present study is distinct from and complementary to both: rather than demonstrating detection performance, it provides a physiological interpretable, feature-level account of how single-site toe PPG morphology changes with PAD severity. Specifically, we (i) decompose the pulse into localized timing, slope, and area features and show that PAD-related information is segment-specific; (ii) report negative findings, identifying features (e.g. raw amplitude) that do not carry PAD-related information under typical acquisition conditions; and (iii) characterize the discriminative behavior of individual features across two clinical sites. By grounding feature behavior in vascular physiology, this work establishes a feature-level reference framework that motivates and supports model development.

By systematically describing which PPG features reflect PAD related hemodynamic changes, this work aims to clarify the physiological basis of PPG waveform alterations in PAD. These insights establish a feature-level foundation for future studies that seek to use PPG signals for accessible, interpretable assessments of PAD.

## II. Results

### A. Dataset Representativeness After Exclusions

After signal-quality screening and exclusions, the combined dataset from two clinical sites consisted of 5,282 toe PPG signals from 2,599 unique patients (1,727 patients at La Jolla and 960 at Hillcrest; 88 patients visited both sites and counted once at the patient level, with recordings contributed to each site’s analyses; see Section E for a sensitivity analysis). 33.9% of recordings were excluded. To assess whether exclusions introduced bias, we compared demographics, comorbidities, and ABI severity between included and excluded recordings at each site (Table 1). Demographic and comorbidity composition was well preserved after exclusion at both sites, with no consistent differences across age, sex, race, ethnicity, or major comorbidities. ABI severity distributions were similar between included and excluded recordings at Hillcrest and modestly shifted toward higher ABI among included recordings at La Jolla. Overall, exclusions did not introduce systemic demographic bias and largely preserved representation across the full severity range, supporting the validity of subsequent feature-level analyses. Full severity-stratified cohort characteristics are reported in our companion modeling work [20].

**TABLE 1:** Dataset Representativeness After Signal-Quality Exclusion. Included Recordings Produced a Clean Representative Pulse and Entered Feature Analysis; Excluded Recordings Were Removed for Signal Quality. Demographics and Comorbidities are Summarized Per Patient; Ankle-Brachial Index (ABI) Severity Per Recording (leg). Percentages are Computed Within Each Column. *p* Values Compare Included Vs. Excluded Within Each Site (Mann–Whitney *U* for Age and ABI; $\chi ^{2}$ for Categorical Variables, One Omnibus Test Per Variable)

	**La Jolla**			**Hillcrest**		
**Characteristic**	Incl.	Excl.	*p*	Incl.	Excl.	*p*
*Patient-level*						
N patients	1727	664	–	960	165	–
Age, median (IQR), y	72 (63–80)	73 (63–80)	0.513	68 (60–77)	70 (61–76)	0.515
Female, %	44.9	40.5	0.057	41.7	35.8	0.180
Diabetes, %	38.3	30.7	***< *0.001**	45.5	43.6	0.715
Hypertension, %	76.5	75.0	0.477	81.5	80.6	0.880
CAD, %	34.0	34.0	1.000	33.9	35.2	0.813
*Race* (omnibus *p*)						
White, %	66.5	69.6	0.347	58.2	58.2	0.596
Other/Mixed, %	17.8	14.6		25.1	21.8	
Black, %	4.2	3.5		7.7	9.1	
Asian, %	10.0	10.8		5.8	8.5	
Unknown, %	1.6	1.5		3.1	2.4	
*Ethnicity* (omnibus *p*)						
Non-Hispanic, %	81.5	86.0	**0.035**	71.7	73.9	0.772
Hispanic, %	15.8	11.9		25.0	22.4	
Unknown, %	2.7	2.1		3.3	3.6	
*Recording-level (legs)*						
N recordings	3457	2108	–	1825	595	–
Median ABI	0.976	1.032	***< *0.001**	1.000	1.000	0.615
IQR ABI (Q1–Q3)	0.74–1.09	0.89–1.12		0.82–1.11	0.80–1.13	
ABI *≤*0.50, %	6.6	5.4	***< *0.001**	4.8	7.9	**0.040**
ABI 0.51–0.70, %	15.2	8.9		11.6	11.9	
ABI 0.71–0.90, %	17.5	12.1		15.9	13.8	
ABI 0.91–1.00, %	16.2	15.8		18.5	16.8	
ABI 1.01–1.40, %	44.5	57.8		49.2	49.6	

^0^C

AD, coronary artery disease; IQR, interquartile range.

Recordings excluded: 2108/5565 (37.9%) at La Jolla, 595/2420 (24.6%) at Hillcrest. The *>*1.40 (non-compressible) bin is excluded from the ABI comparison. Omnibus *p* for race/ethnicity is shown once per block. Bold indicates *p< 0.05*.

**TABLE 2:** Feature-Level Discrimination of Ankle-Brachial Index (ABI)-Defined Peripheral Artery Disease (PAD). Operating Thresholds Were Selected on the La jolla Cohort by Maximizing Youden’s *J* and Applied Unchanged to Hillcrest for Validation. Point Estimates are Shown With 95% Patient-Level Bootstrap Confidence Intervals. Panel (a) Reports Discrimination of PAD (ABI *≤* 0.90) Versus No PAD; panel (b) Reports Sensitivity Across Cumulative Disease-Severity Tiers At the Same Fixed Threshold. Positive Predictive Value (PPV) Reflects the High PAD Prevalence of This Referral Cohort.

(a) Discrimination of PAD (ABI *≤* 0.90) Vs. No PAD									
		**Sensitivity**		**Specificity**		**PPV**		**NPV**	
Feature	Cohort	Est.	(95% CI)	Est.	(95% CI)	Est.	(95% CI)	Est.	(95% CI)
Time to 50% on falling edge	La Jolla$^{\dagger }$	0.808	(0.787–0.832)	0.686	(0.665–0.707)	0.625	(0.600–0.650)	0.847	(0.827–0.866)
		Hillcrest$^{\ddagger }$	0.873	(0.844–0.899)	0.641	(0.611–0.670)	0.537	(0.500–0.574)	0.913	(0.892–0.933)
Systolic area under the curve	La Jolla$^{\dagger }$	0.829	(0.808–0.848)	0.658	(0.635–0.680)	0.610	(0.585–0.636)	0.856	(0.838–0.874)
		Hillcrest$^{\ddagger }$	0.844	(0.811–0.874)	0.627	(0.595–0.656)	0.519	(0.481–0.553)	0.894	(0.870–0.916)
(b) Sensitivity by Cumulative Severity Tier, Among PAD Legs At the Fixed Threshold.									
		ABI *≤* 0.90		ABI *≤* 0.70		ABI *≤* 0.50			
Feature	Cohort	Est.	(95% CI)	Est.	(95% CI)	Est.	(95% CI)		
*PAD legs, *n* (La Jolla / Hillcrest)*		1356 / 589		752 / 299		228 / 87			
Time to 50% on falling edge	La Jolla$^{\dagger }$	0.808	(0.787–0.832)	0.903	(0.881–0.924)	0.939	(0.906–0.968)		
		Hillcrest$^{\ddagger }$	0.873	(0.844–0.899)	0.943	(0.916–0.968)	0.989	(0.961–1.000)$^{\ast }$		
Systolic area under the curve	La Jolla$^{\dagger }$	0.829	(0.808–0.848)	0.890	(0.867–0.912)	0.912	(0.876–0.950)		
		Hillcrest$^{\ddagger }$	0.844	(0.811–0.874)	0.926	(0.894–0.956)	0.966	(0.924–1.000)$^{\ast }$		

^0^C

I, confidence interval; NPV, negative predictive value; PPV, positive predictive value.

$^{\dagger }$Derivation cohort (threshold selected here). $^{\ddagger }$Validation cohort (threshold frozen from La Jolla). $^{\ast }$Upper CI bound at 1.000 reflects the small number of legs in the most severe tier (bootstrap saturation), not perfect sensitivity.

Severity tiers are cumulative and nested (*≤*0.90 *⊃* *≤*0.70 *⊃**≤*0.50); sensitivity is computed among PAD legs only, so the positive class is never redefined and PPV/specificity are unaffected.

Because not all patients contributed analyzable signals from both lower extremities, we additionally evaluated whether leg-level inclusion biased severity representation. ABI distributions among patients contributing only one leg were similar to those of the overall cohort (Supplementary Fig. 1).

**Figure 1.**
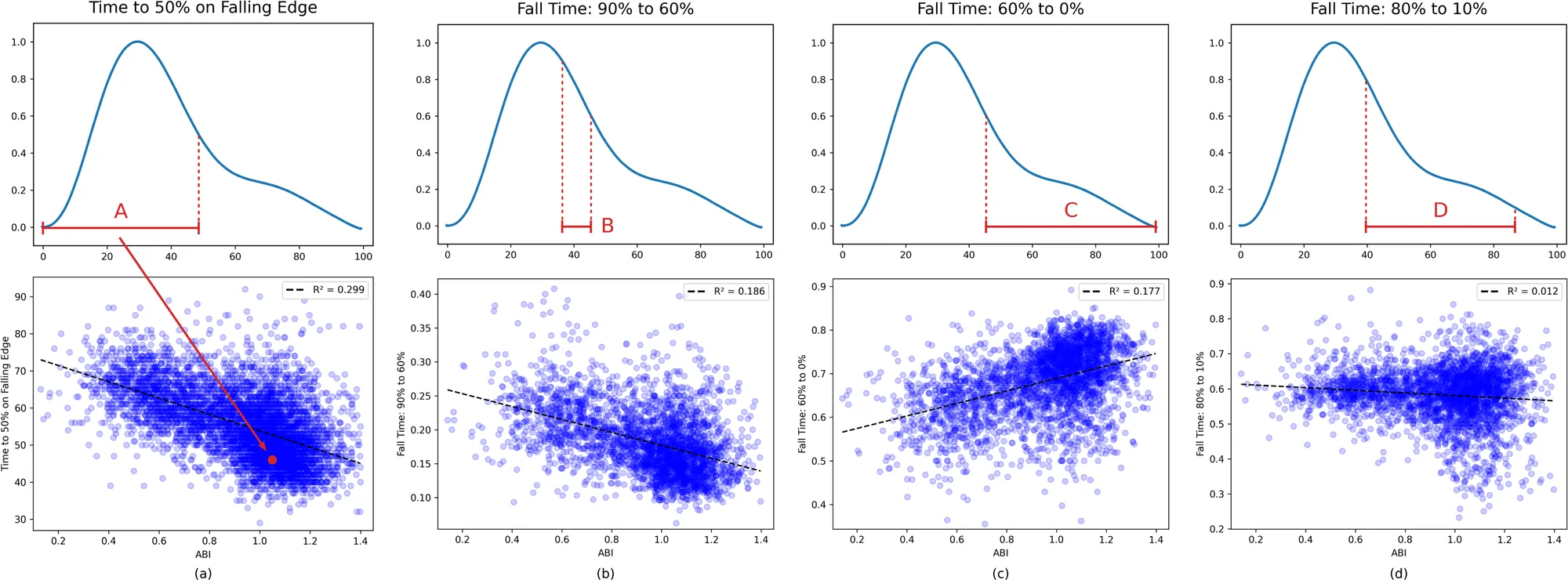
Falling-edge timing features are associated with ankle-brachial index (ABI) in a segment-specific manner (5,282 signals, N=2,599). Top row: each feature (red) defined on a representative normalized toe photoplethysmography (PPG) pulse. Bottom row: each feature plotted against ABI per leg for the combined dataset, with linear fit and $R^{2}$. The red marker and arrow in (a) locate the example pulse’s feature value within the population. (a) Time to 50% amplitude on the falling edge, measured as absolute time from pulse onset (feature A), is negatively associated with ABI ($R^{2}=0.299$, *p< 0.0001*). (b) Early-decay 90-60% segment duration (feature B) is negatively associated ($R^{2}=0.186$, *p< 0.0001*) and (c) late-decay 60-0% segment duration (feature C) is positively associated ($R^{2}=0.177$, *p< 0.0001*), showing opposing localized effects. (d) The aggregate 80-10% segment (feature D) shows no association ($R^{2}=0.012$, *p< 0.0001*), indicating that broad timing measures obscure these localized changes. Features B-D are relative segment durations; feature A is an absolute onset-referenced time.

### B. Falling-Edge Timing is Associated With ABI in a Segment-Specific Manner

We evaluated how the temporal distribution of the PPG falling edge relates to PAD severity as quantified by ABI (Fig. 1, features A-D). Time to 50% amplitude on the falling edge was negatively associated with ABI ($R^{2}=0.299$, *p< 0.0001*), with lower ABI corresponding to delayed waveform progression (Fig. 1(a)). Decomposing the falling edge into segments revealed opposing, localized associations: the early-decay 90–60% interval was negatively associated with ABI ($R^{2}=0.186$, Fig. 1(b)), while the late-decay 60-0% interval was positively associated ($R^{2}=0.177$, Fig. 1(c)). A broad aggregate measure spanning 80-10% showed no association ($R^{2}=0.012$, Fig. 1(d)), indicating that aggregated timing metrics obscure the localized, opposing changes that segment-specific features capture.

### C. Localized Slope and Integrated Area Features Reflect the Same Temporal Shift

Slope and area features captured the same underlying shift (Fig. 2, features E-H). Slope evaluated at 37% of normalized pulse duration was negatively associated with ABI ($R^{2}=0.256$, *p< 0.0001*, Fig. 2(a)), while slope at 68% was positively associated with ABI ($R^{2}=0.201$, *p< 0.0001*, Fig. 2(b)), consistent with a temporal displacement of waveform progression with increasing severity. Slope features evaluated near the beginning, midpoint, or end of the waveform showed minimal association with ABI, indicating that slope is informative only at time points sensitive to this temporal misalignment. Both systolic area ($R^{2}=0.278$, *p< 0.0001*, Fig. 2(c)) and total pulse area ($R^{2}=0.239$, *p< 0.0001*, Fig. 2(d)) were inversely associated with ABI, with larger normalized areas at lower ABI, indicating that the timing shift accumulates across both the systolic and overall pulse. Consistent trends were observed when the two sites were analyzed separately across all presented features (Supplementary Figs. 2– 5).

**Figure 2.**
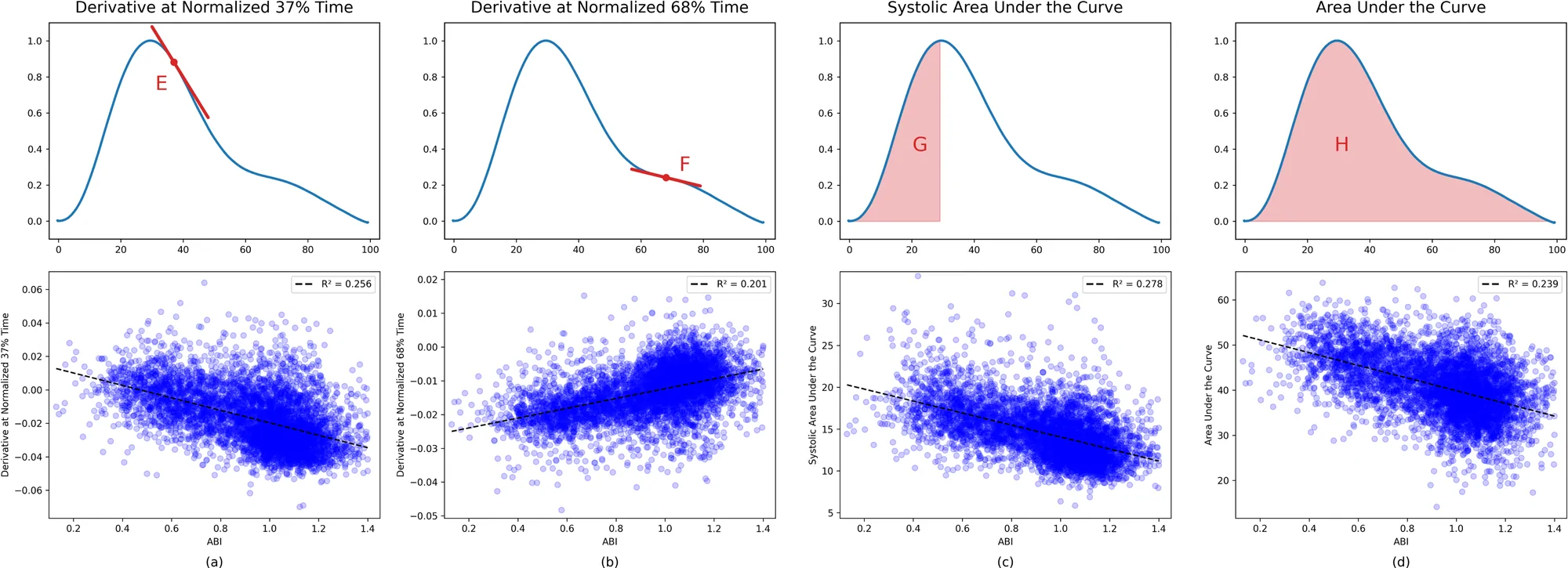
Localized slope and integrated area features reflect a common temporal shift (5,282 signals, N=2,599). Top row: each feature (red) defined on a representative normalized toe photoplethysmography (PPG) pulse. Bottom row: each feature plotted against ankle-brachial index (ABI) per leg for the combined dataset, with linear fit and $R^{2}$. (a) Slope at 37% normalized pulse duration (feature E) is negatively associated with ABI ($R^{2}=0.256$, *p< 0.0001*) and (b) slope at 68% (feature F) is positively associated ($R^{2}=0.201$, *p< 0.0001*), consistent with a temporal displacement of waveform progression with increasing severity. (c) Systolic area under the curve ($R^{2}=0.278$, *p< 0.0001*, feature G) and (d) total pulse area under the curve ($R^{2}=0.239$, *p< 0.0001*, feature H) are both inversely associated with ABI, indicating the shift accumulates across the systolic and overall pulse. Larger normalized area reflects prolonged morphology, not increased blood volume.

### D. Severity-Graded Morphological Progression

Averaging normalized waveforms within ABI strata showed a graded morphological progression with severity (Fig. 3(a)): lower ABI corresponded to a later peak and slower, more prolonged decay. The contrast between severe PAD (ABI *≤* 0.5) and normal (1.0 *< * ABI *≤* 1.4) waveforms remained distinct relative to within-group variability (*±*1 standard deviation, Fig. 3(b)).

**Figure 3.**
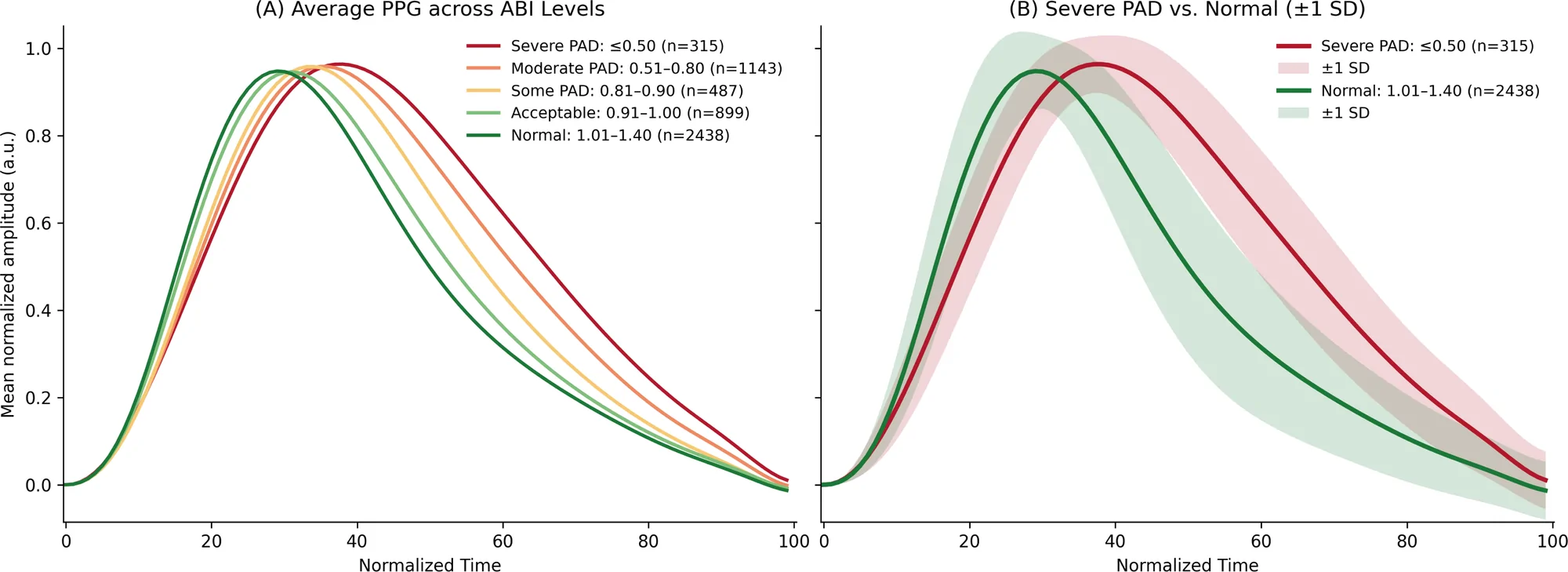
Average toe photoplethysmography (PPG) waveform morphology across ankle-brachial index (ABI) severity strata (pooled cohorts from combined dataset of 5,282 signals, N=2,599). (a) Mean normalized waveform for each ABI stratum, showing a graded progression toward a later peak and slower, more prolonged decay with decreasing ABI. (b) Mean waveforms for severe peripheral artery disease (PAD) (ABI *≤* 0.5) and normal (1.0 *< * ABI *≤* 1.4) with *±*1 standard deviation (SD) bands, showing the contrast persists relative to within-group variability. n per stratum as labeled.

### E. Feature-Level Discrimination of ABI-Defined PAD

To characterize the discriminative behavior of individual features at a clinical threshold, we evaluated two features (time to 50% on falling edge and systolic area under the curve) as a single-threshold classifier of ABI-defined PAD (ABI *≤* 0.90). Thresholds were selected on La Jolla by maximizing Youden’s J and applied unchanged to Hillcrest. We emphasize that this analysis characterizes the standalone behavior of individual features and does not constitute a combined predictive model; multi-feature score development and model-based PAD detection are addressed in separate work [20].

The two features characterized here (time to 50% on falling edge and systolic area under the curve) were selected prior to this analysis based on physiological rationale and their consistent association with ABI in our prior work [19], and represent distinct feature classes (falling-edge timing and integrated area). They were not selected based on their derivation-validation transfer performance.

Both headline features discriminated PAD with moderate, cross-cohort-stable performance (Table (2a)): time to 50% achieved sensitivity 0.81 / specificity 0.69 in La Jolla and 0.87 / 0.64 in Hillcrest; systolic area achieved 0.83 / 0.66 and 0.84 / 0.63, respectively. Positive predictive values (0.52–0.63) reflect the high PAD prevalence of this referral cohort and would be expected to differ in a lower-prevalence screening population; negative predictive values were high (0.85–0.91). That a threshold fixed on one site retained its operating characteristics at an independent site, acquired with different equipment and personnel, indicates that these feature-level relationships are not specific to a single acquisition setting.

Eighty-eight patients contributed recordings at both sites (3.4% of the cohort). To confirm that this overlap did not inflate the validation performance, we repeated the Hillcrest analysis after excluding all recordings from these patients (Supplementary Table 1). All operating characteristics remained within 0.02 of the primary values, with sensitivity, specificity, and negative predictive value changing by less than 0.02 and positive predictive value shifting in proportion to the reduced PAD prevalence in the disjoint subset. Severity-tier sensitivity was similarly unchanged.

Discriminative performance strengthened with disease severity. At the same fixed threshold, sensitivity among PAD legs increased monotonically with disease severity (Table (2b)): for time to 50%, sensitivity rose from 0.87 among all PAD legs to 0.94 among severe legs (ABI *≤* 0.70) and 0.99 among the most severe legs (ABI *≤* 0.50) in Hillcrest, with the same pattern in La Jolla (0.81, 0.90, 0.94) and for systolic area (0.84, 0.93, 0.97 in Hillcrest and 0.83, 0.89, 0.91 in La Jolla). This severity-graded behavior is consistent with more extreme cases lying further from the diagnostic threshold, and suggests that borderline cases near ABI 0.90 contribute to the moderate discrimination at the full threshold. We note that this analysis reports sensitivity within nested severity tiers at a fixed threshold and does not directly quantify discrimination between severity categories.

### F. Amplitude Features Show No Association With ABI

We evaluated multiple amplitude-based PPG features to assess whether signal magnitude is associated with PAD severity as quantified by ABI (Fig. 4). Raw PPG baseline-to-peak amplitude exhibited substantial variability and did not demonstrate a meaningful relationship with ABI ($R^{2}=0.047$, Fig. 4(a)). Because absolute amplitude depends on acquisition hardware and sensor configuration and raw scaling factors were only accessible at La Jolla, these amplitude analyses were restricted to a single clinical cohort (La Jolla).

**Figure 4.**
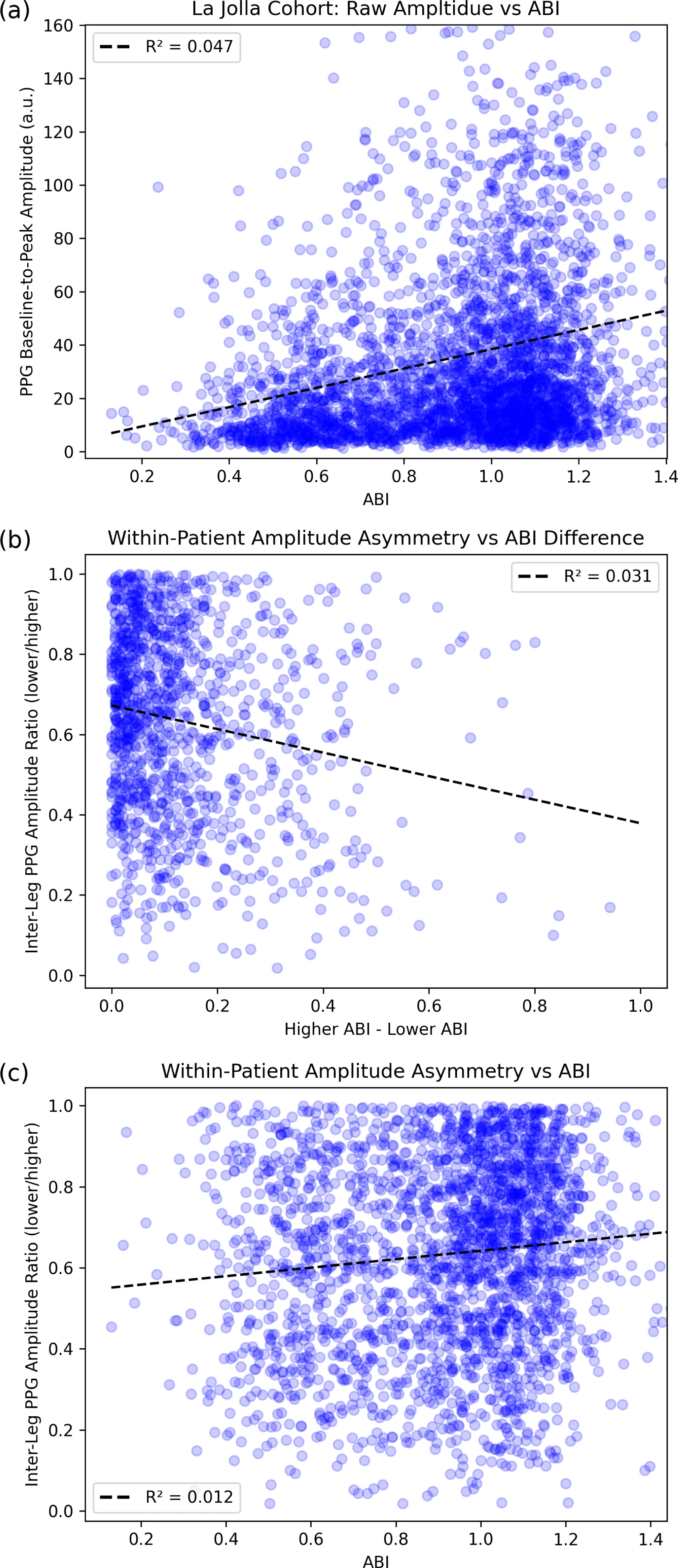
Amplitude-based photoplethysmography (PPG) features show no meaningful association with ankle-brachial index (ABI) (La Jolla cohort, 3,457 signals, N=2,226). (a) Raw baseline-to-peak amplitude versus ABI ($R^{2}=0.047$). (b) Within-patient inter-leg amplitude ratio versus inter-leg ABI difference ($R^{2}=0.031$). (c) Within-patient inter-leg amplitude ratio versus ABI ($R^{2}=0.012$). Dashed lines show linear fits.

To account for within-person variability, we next examined relative amplitude differences between lower extremities and compared these to inter-leg ABI differences. Relative amplitude differences did not show a structured relationship with ABI differences between legs ($R^{2}=0.031$, Fig. 4(b)) or demonstrate clustering or separation across ABI values indicative of PAD severity ($R^{2}=0.012$, Fig. 4(c)).

Together, these analyses indicate that amplitude-based PPG features do not exhibit meaningful associations with ABI in this dataset.

## III. Discussion

Prior PPG-based approaches to PAD have largely fallen into two categories: machine-learning classifiers that achieve discrimination at the cost of physiological interpretability [14], [15], and smaller descriptive studies using multi-site pulse comparisons that interpret morphology but rely on bilateral comparisons and do not characterize features systematically at scale [16], [17], [18]. This study connects these strands by quantifying the discriminative behavior of interpretable, single-site toe PPG features in a large multi-site cohort, establishing which waveform characteristics encode PAD-related information and which do not, and showing that this behavior is severity-graded and transfers across acquisition settings. This feature-level grounding is a prerequisite for, rather than an alternative to, model development: it identifies the features a model should rely on and provides a physiological basis for trusting its outputs across populations and devices.

The central finding is that PAD severity is reflected in a temporal shift in PPG pulse morphology, most prominently reflected in delayed progression of the falling edge, rather than in signal magnitude. This shift is segment-specific with early and late portions of the falling edge exhibiting opposing associations with ABI, while a broad aggregate measure spanning the decay shows none. This indicates that aggregated metrics can mask localized changes arising from a common underlying displacement of waveform progression. Localized slope features are informative only at intermediate locations sensitive to this misalignment, and integrated area features show the shift accumulates across the systolic and overall pulse. These observations are consistent with established clinical understanding of PAD as a condition of distal arterial obstruction and increased stiffness, which alter pulse transmission and decay [16], [21], [22], [23].

We note that this physiological alignment is interpreted as consistency with known vascular mechanisms, not as direct mechanistic proof. Our evidence is feature-ABI association benchmarked against a clinical reference standard where the mapping of these associations to specific physiological processes is grounded in prior literature rather than independently demonstrated here. We have framed these interpretations accordingly.

The reported associations are statistically significant but modest in magnitude ($R^{2}=0.18-0.30$). We interpret these effect sizes in context. They are computed across the full ABI range over several thousand measurements, benchmarked against a reference standard (ABI) with its own measurement variability, in a retrospective cohort without per-analysis signal curation. In this setting, a single feature explaining a quarter to a third of the variance represents a consistent and physiologically coherent association. Moreover, the feature-level discrimination analysis shows that these same features discriminate clinically defined PAD with moderate, cross-cohort-stable performance, and that their sensitivity increases monotonically with severity. Modest full-range correlation and stronger sensitivity at more extreme ABI values are therefore not in tension as borderline cases near the diagnostic threshold likely contribute to the moderate correlation, while cases further from the threshold are detected more reliably at the same operating point. Accordingly, we describe these features as informative and physiologically grounded, and characterize the framework as a feature-level reference, rather than claiming that individual waveform morphology comprehensively captured PAD severity.

The absence of association between raw PPG amplitude and ABI provides methodological context for these findings. Amplitude did not vary systematically with PAD severity, consistent with its dependence on acquisition conditions and individual tissue characteristics such as sensor placement, contact pressure, and toe properties, which introduce variability unrelated to disease. This negative result is itself informative: it supports the use of amplitude normalization and motivates the focus on shape and timing features, which remained consistently associated with ABI despite this variability. Reporting features that do not carry PAD-related information, alongside those that do, is part of establishing a reliable feature-level reference.

These results do not position, nor intend to position, PPG as a replacement for ABI. ABI remains the reference standard against which our features were benchmarked, and our analysis inherits its known limitations, including reduced sensitivity in non-compressible vessels and in diabetic or renal disease [24], [25]. Rather, the consistent, physiologically grounded relationships between toe PPG morphology and ABI-defined PAD support a complementary role. Toe PPG is the physiologically appropriate site for lower-extremity PAD, as the disease affects perfusion in the legs and feet; whether comparable information is recoverable from finger- or wrist-based PPG, anatomically remote from the affected territory, is an important open question for accessible screening and a direction for future work.

Several limitations should be considered. All analyses were retrospective and relied on ABI as the reference standard. Although signal-quality exclusions did not introduce observable demographic bias and largely preserved representation across the full severity range, a substantial proportion of recordings was excluded, motivating more robust signal-quality methods. We did not formally assess skin tone or optical tissue properties, which given known disparities in PAD outcomes [3], [8], warrant dedicated study under controlled acquisition conditions. Features were derived from short four-second toe PPG segments at two campuses of a single health system; broader validation across institutions, longer acquisitions, and consumer-grade devices will be necessary to confirm generalizability. The validation of a combined predictive model addressed in our separate work [20] and prospective evaluation remain necessary next steps toward clinical application.

## IV. Conclusion

This study provides a large-scale, multi-site, feature-level characterization of how toe PPG morphology relates to PAD severity. PAD-related information was concentrated in the timing and shape of the pulse, specifically in the delayed and prolonged falling-edge progression, segment-specific and opposing decay timing, localized slope changes at intermediate waveform locations, and increased normalized pulse area. Raw amplitude carried no reliable information. These findings establish a physiologically interpretable feature-level reference framework that supports and is complementary to model-based PAD detection.

## V. Materials and Methods

This study used ABI measurements as the clinical reference standard for quantifying PAD severity. Clinical and waveform data were obtained retrospectively from the La Jolla and Hillcrest campuses within the University of California San Diego Health System. All procedures were approved by the Institutional Review Board of the University of California San Diego (IRB Approval #809658). PPG recordings were collected at the big toe as part of routine, clinically-indicated ABI testing. All PPG recordings were four seconds in duration. Raw waveforms were screened using predefined signal quality criteria; samples were excluded if the segment contained unfilterable motion artifacts or waveform clipping, nonphysiological dominant frequency components, or missing information needed for ABI calculation. Recordings with ABI *>* 1.40 (suggestive of non-compressible arteries) were also excluded. ABI severity categories were defined using a right-closed convention throughout (i.e., each stratum includes its upper bound), and the same convention was applied at the PAD threshold (ABI *≤* 0.90). Following exclusions, the final cohort consisted of 5,282 toe PPG signals from N = 2,599 unique patients across the combined dataset, where each toe PPG signal has a corresponding ABI measurement. Representativeness of the included versus excluded recordings is reported in Table 1.

All waveform data were preprocessed with noise removal and independent normalization of amplitude and time scales, from which a single representative pulse was extracted per recording. Amplitude-based features were computed from raw (non-normalized) waveforms. All morphology features were computed from normalized pulses, as depicted in Fig. 1 and 2. Feature-level associations with ABI were evaluated by linear regression, with *p< 0.0001* considered significant. To visualize morphological progression with severity, normalized pulses were pooled across sites and averaged within ABI strata (Fig. 3).

To characterize the discriminative behavior of individual features at a clinical decision threshold, each feature was evaluated as a single-threshold classifier of ABI-defined PAD. For each feature, an operating threshold was selected on the La Jolla cohort by maximizing Youden’s J and applied unchanged to the Hillcrest cohort for independent validation. Sensitivity, specificity, and predictive values were computed at the fixed threshold, with sensitivity additionally stratified by cumulative severity; 95% confidence intervals were estimated by patient-level bootstrap. Full details of the study population, preprocessing, feature extraction, and analysis are provided in the Supplementary Materials.

## VI. Supplementary Material

Please visit the supplementary materials accompanying this article for additional results figures and expanded methodological information. For results, the supplement includes figures showing the consistency of feature associations across the two clinical sites as well as a patient-disjoint sensitivity analysis for the feature operating thresholds. These materials also provide detailed context beyond the brief methods summary here, including dataset details, signal preprocessing steps, and pulse extraction procedures.

Supplementary Materials
