# Supplementary material for: Physiologically Interpretable Photoplethysmography Features Associated With Peripheral Artery Disease
Source: IEEE Open J Eng Med Biol. 2026 Sep 8;7:308–15. doi: 10.1109/OJEMB.2026.3732080 (PMC13667292; doi:10.1109/OJEMB.2026.3732080)
Supplement: Supplementary file 1 [file supp1-3732080.pdf]

Supplementary Materials

Physiologically Interpretable Photoplethysmography Features Associated with Peripheral Artery Disease

Ava J. Fascetti*, Mustafa H. Naguib, Elsie G. Ross, Chris Longhurst, Pam R. Taub, Shamim Nemati, Edward J. Wang, Mattheus Ramsis

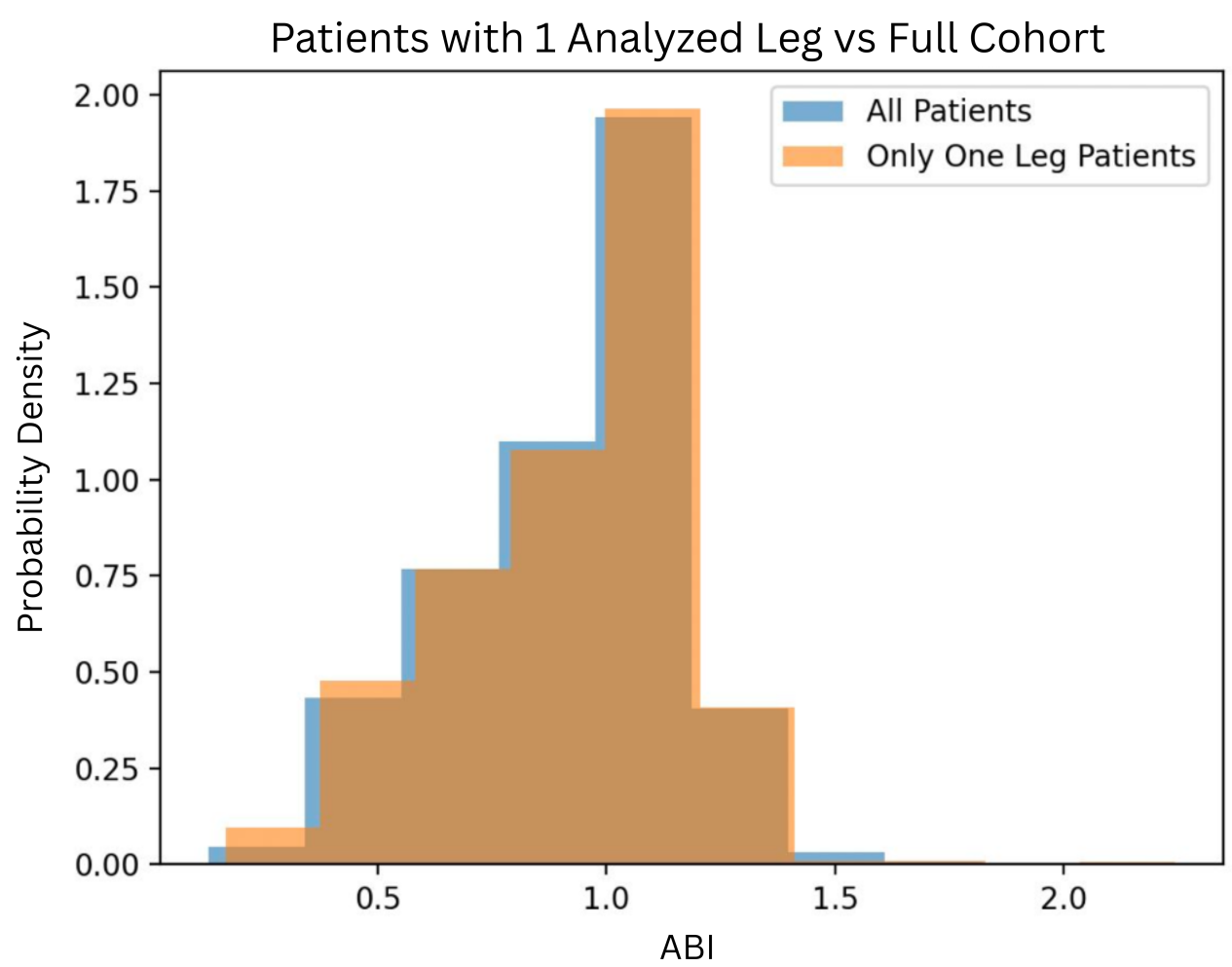

Figure 1: Normalized distribution of ankle-brachial index (ABI) values among the full cohort (blue) compared to patients with only one leg in the analysis (orange).

THESE Supplementary Materials contain additional results figures and the details of the Materials and Methods section of the main manuscript.

A. Supplemental Results

To further support the dataset representativeness analysis, we include here in Supplemental Fig. 1 evidence that patients with only one leg in the dataset share a similar ankle-brachial index (ABI) distribution as the full dataset. Patients have one leg in the dataset (rather than both) for a variety of reasons, including because they have an amputation. To explicitly determine whether patients with a single leg in the analysis biases the dataset to those who may already have peripheral artery disease (PAD), we compare the severity levels to the full cohort. As no differences are detected, this suggests we do not bias our dataset by using these signals analogously as well as supports a leg-level analysis across the dataset.

To show that the associations between photoplethysmography (PPG) pulse morphology and ABI are consistent across our two clinical sites, we include here variations of the main manuscript results, where datapoints are color-coordinated for acquisition site. Specifically, in Supplemental Fig. 2, we show the segment-specific associations between falling edge timing features and ABI, where La Jolla collected signals are shown in blue and Hillcrest collected signals are shown in orange. In Supplemental Fig. 3, we do the same for the delayed fall timing feature. In Supplemental Fig. 4, we show the localized slope associations, and in Supplemental Fig. 5 we present the integrated pulse area features.

To confirm that the 88 patients contributing recordings at both clinical sites did not inflate the validation performance reported in the main manuscript, we include here in Supplemental Table I a patient-disjoint sensitivity analysis of the feature-level discrimination results. Hillcrest recordings from these overlapping patients were excluded and the analysis was rerun using the same operating thresholds derived on La Jolla. All operating characteristics remained within 0.02 of the values in main manuscript Table II, with the exception of positive predictive value, which shifted in proportion to the modestly reduced PAD prevalence in the patient-disjoint Hillcrest subset. Severity-tier sensitivity was similarly unchanged. This supports that the cross-site validation reported in the main manuscript is not driven by shared patients across acquisition settings.

B. Supplemental Materials and Methods

Portions of this section are derived from the original conference paper [19].

This retrospective data analysis study used data collected from adult patients who presented for a clinically-indicated ABI assessment at the University of California San Diego La Jolla or Hillcrest locations between 2020 and 2025. As part of a routine ABI assessment, PPG signals are collected at each toe, and blood pressure (BP) readings are taken at various sites of the body. Raw toe PPG waveform signals were extracted from the vascular laboratory database at each site. Note that two different company's equipment is used at each location, and as such the PPG capturing mechanisms are different. All PPG samples used in analysis are four seconds long. For each leg for each patient, an ABI value is calculated using the collected BP readings during the assessment. We used sensitive criteria for ABI, and as such, the lower blood pressure between the posterior tibial and dorsalis pedis was used in combination with the maximum left or right brachial blood pressure. Demographic data including age, sex, and race were extracted from linked electronic health records (EHRs). All procedures were approved by the institutional review board of the University of California San Diego (IRB Approval #809658).

The population of patients with known compressible ABI in La Jolla is N=2,391 and in Hillcrest is N=1,125. These datasets combine to give N=3,428 unique patients for analysis (some are shared across dataset). Demographics and PAD severity across these sites are reported in the main manuscript in Table I. It is

Table I: Sensitivity analysis of feature-level discrimination excluding the 88 patients with recordings at both sites. Hillcrest recordings from these patients (189 recordings) were excluded; La Jolla derivation is unchanged. Format matches main text Table II. All primary operating characteristics remained within 0.02 of main text Table II values; PPV shifted in proportion to the reduced PAD prevalence in the disjoint subset (29.5% vs. 32.3%).

(a) Discrimination of PAD (ABI $\leq$ 0.90) vs. no PAD

Feature	Cohort	**Sensitivity** Est. (95% CI)	**Specificity** Est. (95% CI)	**PPV** Est. (95% CI)	**NPV** Est. (95% CI)
Time to 50% on falling edge	La Jolla†	0.808 (0.787–0.832)	0.686 (0.665–0.707)	0.625 (0.600–0.650)	0.847 (0.827–0.866)
	Hillcrest‡	0.880 (0.850–0.909)	0.648 (0.619–0.680)	0.511 (0.474–0.548)	0.928 (0.911–0.946)
Systolic area under the curve	La Jolla†	0.829 (0.808–0.848)	0.658 (0.635–0.680)	0.610 (0.585–0.636)	0.856 (0.838–0.874)
	Hillcrest‡	0.853 (0.820–0.887)	0.633 (0.604–0.665)	0.493 (0.453–0.529)	0.911 (0.891–0.933)

(b) Sensitivity by cumulative severity tier, among PAD legs at the fixed threshold

Feature	Cohort	ABI $\leq$ 0.90 Est. (95% CI)	ABI $\leq$ 0.70 Est. (95% CI)	ABI $\leq$ 0.50 Est. (95% CI)
PAD legs, n (La Jolla / Hillcrest)		1356 / 482	752 / 241	228 / 68
Time to 50% on falling edge	La Jolla†	0.808 (0.787–0.832)	0.903 (0.881–0.924)	0.939 (0.906–0.968)
	Hillcrest‡	0.880 (0.850–0.909)	0.946 (0.918–0.973)	1.000 (1.000–1.000)*
Systolic area under the curve	La Jolla†	0.829 (0.808–0.848)	0.890 (0.867–0.912)	0.912 (0.876–0.950)
	Hillcrest‡	0.853 (0.820–0.887)	0.934 (0.898–0.963)	0.985 (0.952–1.000)*

CI, confidence interval; NPV, negative predictive value; PPV, positive predictive value.
†Derivation cohort (threshold selected here). ‡Validation cohort (threshold frozen from La Jolla). *Upper CI bound at 1.000 reflects the small number of legs in the most severe tier (bootstrap saturation), not perfect sensitivity.
Severity tiers are cumulative and nested ($\leq$0.90 $\supset$ $\leq$0.70 $\supset$ $\leq$0.50); sensitivity is computed among PAD legs only, so the positive class is never redefined and PPV/specificity are unaffected.

important to note that the demographics vary across clinical site due to the differences in location and typical patient population (e.g., Hillcrest serves a more socioeconomically disadvantaged, urban patient population).

The featurization pipeline is consistent across datasets. First, the signals are preprocessed. The two datasets are sampled at different frequencies. Therefore, both sets of signals are resampled such that it matches the lower resolution of 75 Hz (the higher resolution dataset has 125 Hz). Next, both sets of signals are band-pass filtered such that only frequencies in the range of 0.5 Hz and 8 Hz remain in the signal. This frequency range was selected to filter out artifact noise from breathing or other movements, as well as high frequency noise from sensing equipment.

After filtering, both sets of signals are passed through a data cleaning pipeline to discard signals that are deemed too noisy for appropriate analysis. Signals are discarded if the main frequency components of the signal are outside the physiological expected range for heart rate. Additionally, signals with a corresponding ABI above 1.40 (suggestive of non-compressible arteries), or labeled non-compressible, were excluded. With not all patients having two legs included in the analysis and some having repeat measurements, the total number of signals sent into this pipeline from the unique 3,428 patients is 7,985 signals. Approximately one third of these signals are deemed too noisy for featurization, and as such, 5,282 total PPG signals are used moving forward for analysis from N=2,599 unique patients.

To generate features for each PPG signal, one representative, normalized pulse per four-second PPG signal is generated. Features of amplitude are calculated prior to normalization to preserve the raw morphology of the pulse. All other features are calculated after this pipeline. To select a representative pulse, all complete eligible pulses in the four-second signal are identified and normalized in amplitude (starting amplitude at zero and peak at one) and relative time (interpolated for 100 points). Next, any outliers are removed from consideration. If one eligible pulse remains, that pulse is used as the representative example. If multiple pulses remain after outlier removal, the median pulse is selected. At the end of this pipeline, each toe PPG signal yields one standardized pulse. Features are computed directly from this pulse and compared to each other in this normalized space.

Features were constructed in different general categories including rise features, fall features, derivative features, and various miscellaneous features. Features presented in this analysis are defined graphically in the main manuscript in Fig. 1 and 2. For completeness, we describe them textually here as well. Delayed fall timing to 50% on the falling edge is computed as the relative time between pulse onset and the first sample reaching 50% normalized amplitude on the falling edge. Segment-specific falling edge time is computed as the temporal distance between the first instance of the higher normalized amplitude threshold and the first instance of the lower threshold on the decay. Derivatives of the pulse at specified time points are computed using the first derivative of the signal. Integrated measures of area under the curve (AUC) are computed using the normalized pulses. Systolic AUC is calculated as the integral from pulse onset to the pulse peak and total AUC is calculated across the full pulse duration.

With all features computed, they can be directly compared

against that PPG signal's associated ABI measurement. As such, each data point in the results graphs corresponds to one PPG signal (in other words, one patient's right or left leg). As PAD can manifest in one leg and not the other, it is appropriate to treat each leg as a separate data point, as further described in Supplemental Fig. 1. ABI was treated as a continuous clinical severity scale. Linear regression was used to evaluate the association between individual PPG features and ABI values. Coefficients of determination, R^2 values, were computed for each relationship with a statistical significance of $p < 0.0001$ considered significant. Analyses were also performed separately within each site to confirm robustness.

To assess the discriminative behavior of individual features at a clinical decision threshold, each presented feature was evaluated as a single-threshold classifier of ABI-defined PAD (positive: ABI $\leq$ 0.90). For each feature, an operating threshold and direction were selected on the La Jolla cohort as the values maximizing Youden's J (sensitivity + specificity - 1) over the empirical receiver operating characteristic; this threshold was then held fixed and applied to the Hillcrest cohort as an independent validation. Sensitivity, specificity, positive predictive value, and negative predictive value were computed at the fixed threshold for each cohort. To quantify the discrimination of more severe disease, sensitivity was additionally stratified by cumulative severity tiers (ABI $\leq$ 0.90, $\leq$ 0.70, $\leq$ 0.50) among positive legs at the same fixed threshold; specificity and predictive values were not redefined for these tiers, as the analysis reports recall among progressively more severe subsets rather than a re-thresholded classifier. Because patients could contribute measurements from both legs, 95% confidence intervals were estimated by patient-level bootstrap resampling (1,000 iterations), in which patients were sampled with replacement and all of a sampled patient's legs were retained; the threshold remained fixed across iterations. This analysis characterizes individual features and does not develop or evaluate a multi-feature predictive model.

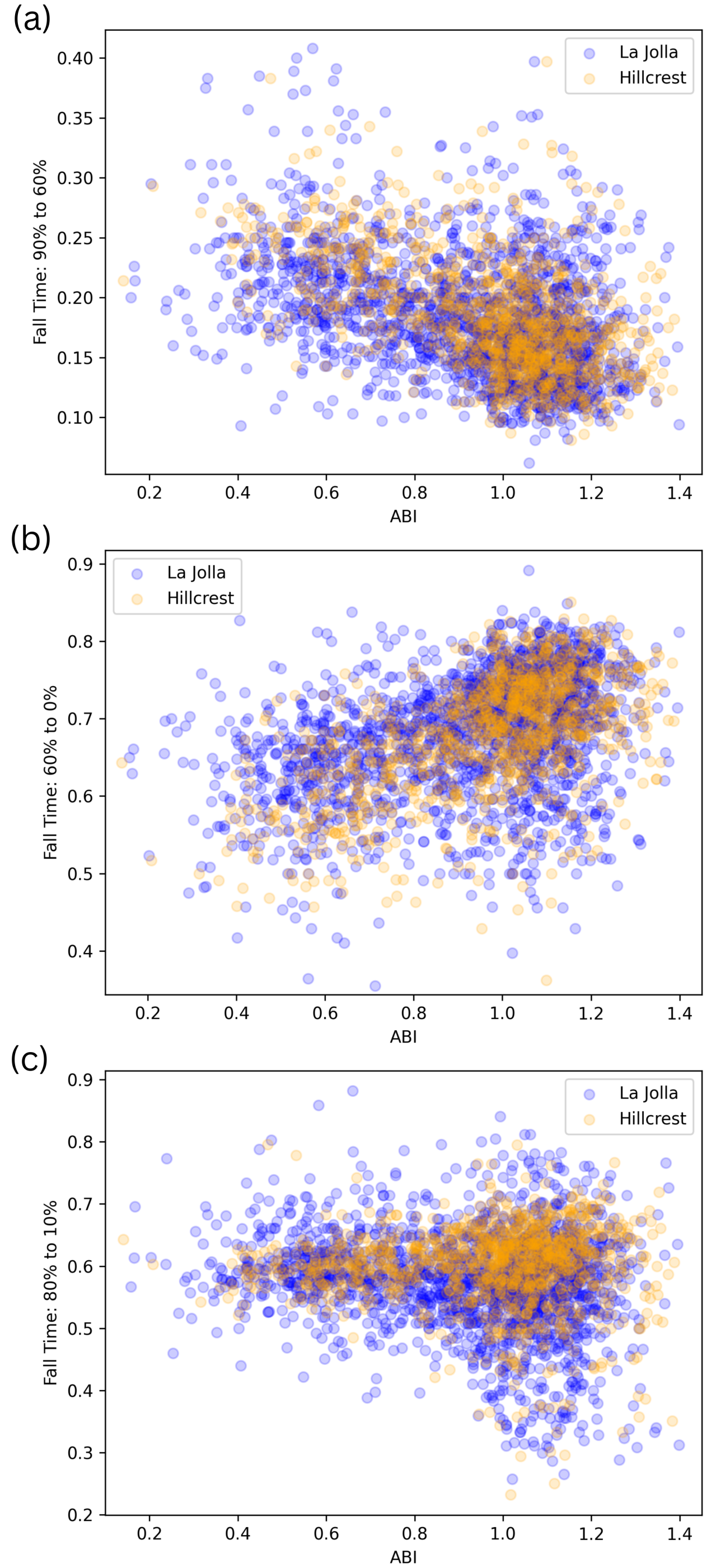

Figure 2: Segment-specific associations between falling edge timing features and ankle-brachial index (ABI) color-coordinated by acquisition site. Scatterplots show ABI versus (a) 90–60%, (b) 60–0%, and (c) 80–10% fall times for the combined dataset with La Jolla signals shown in blue and Hillcrest signals shown in orange.

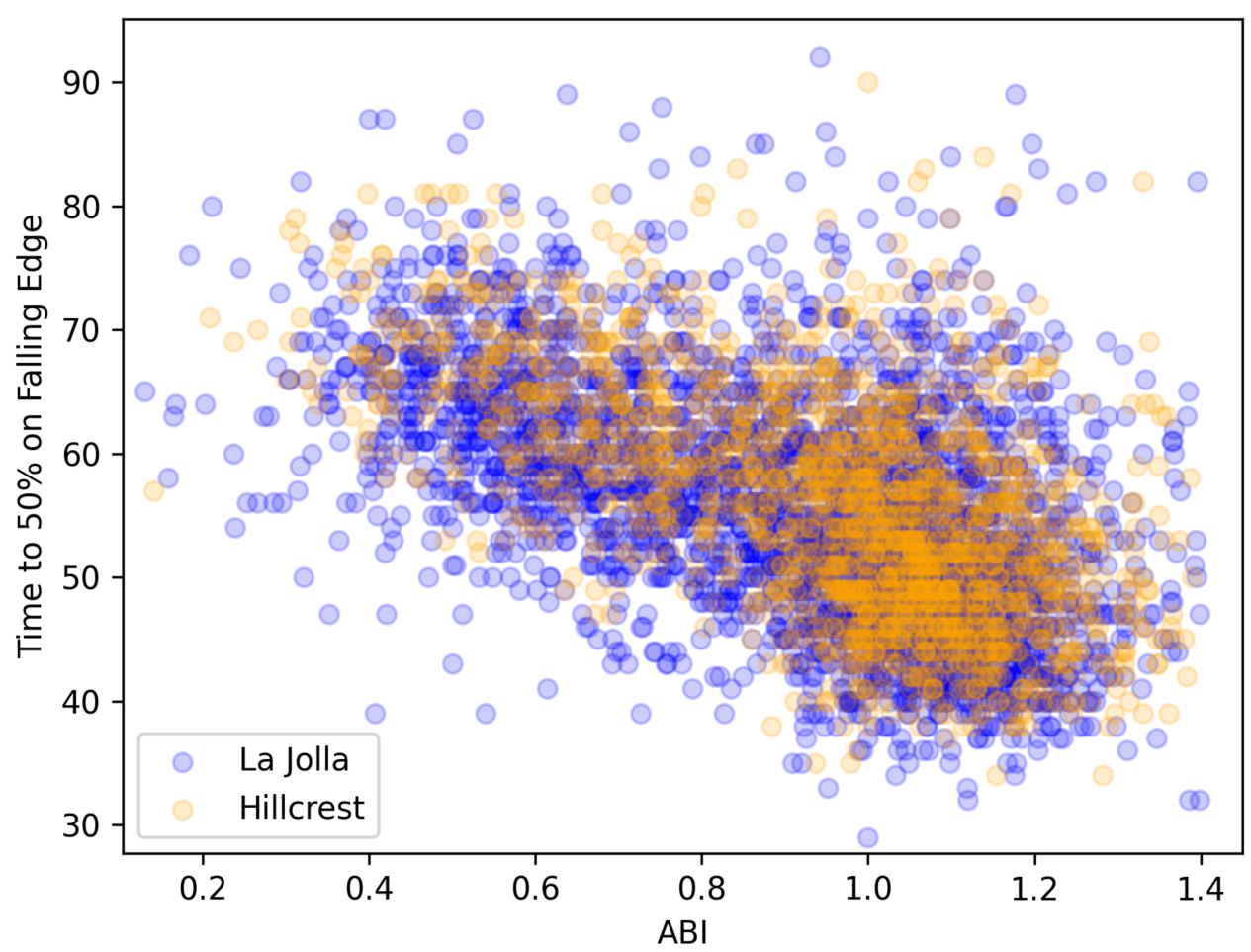

Figure 3: Delayed fall timing relative to pulse onset color-coordinated by acquisition site. Scatterplot shows the relationship between ankle-brachial index (ABI) and time from pulse onset to 50% amplitude on the falling edge for the combined dataset with La Jolla signals shown in blue and Hillcrest signals shown in orange.

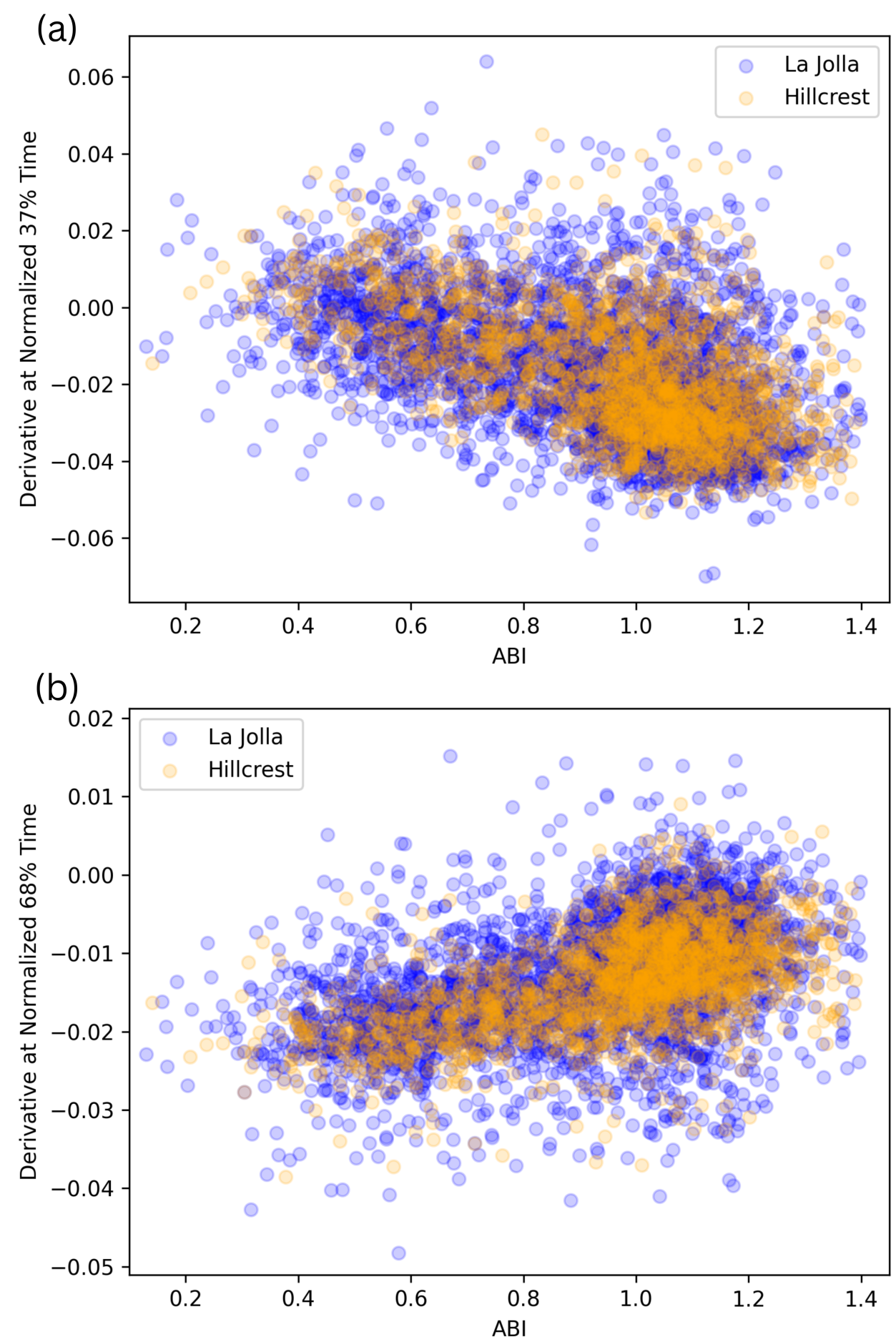

Figure 4: Localized slope associations with ankle-brachial index (ABI) at intermediate waveform locations color-coordinated by acquisition site. Scatterplots show the relationship between ABI and slope features computed at representative intermediate time points along the PPG pulse for the combined dataset of La Jolla (blue) and Hillcrest (orange): (a) slope at 37% of normalized pulse duration and (b) slope at 68% of normalized pulse duration. Percentages are defined from pulse onset (0%) to pulse end (100%).

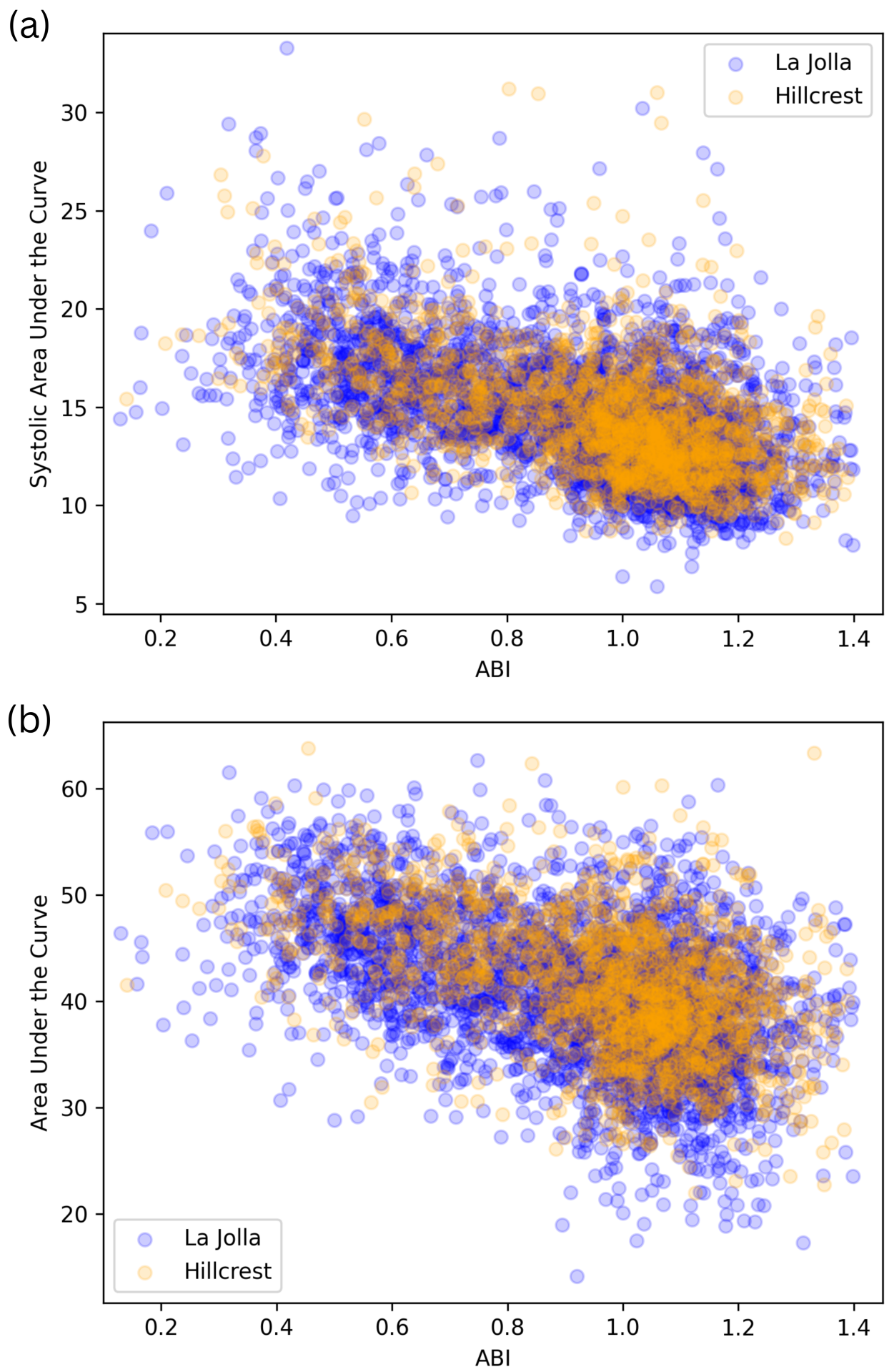

Figure 5: Color-coordinated integrated pulse area features by acquisition site are associated with ankle-brachial index (ABI). Scatterplots show relationships between ABI and (a) systolic area under the curve and (b) total pulse area under the curve for the combined dataset of La Jolla (blue) and Hillcrest (orange).
